# Stabilizing spin spirals and isolated skyrmions at low magnetic field exploiting vanishing magnetic anisotropy

**DOI:** 10.1038/s41467-018-03240-w

**Published:** 2018-03-09

**Authors:** Marie Hervé, Bertrand Dupé, Rafael Lopes, Marie Böttcher, Maximiliano D. Martins, Timofey Balashov, Lukas Gerhard, Jairo Sinova, Wulf Wulfhekel

**Affiliations:** 1 Physikalisches Institut, Karlsruhe Institute of Technology, 76131 Karlsruhe, Germany; 2 Institut für Physik, Johannes Gutenberg Universität Mainz, 55099 Mainz, Germany; 3 Institute of Theoretical Physics and Astrophysics, University of Kiel, 24098 Kiel, Germany; 4 Centro de Desenvolvimento da Tecnologia Nuclear, 31270-901 Belo Horizonte, Brazil; 5 Institute of Nanotechnology, Karlsruhe Institute of Technology, 76128 Karlsruhe, Germany; 6 Institute of Physics, Academy of Sciences of the Czech Republic, Cukrovarnická 10, 162 53 Praha 6, Czech Republic

## Abstract

Skyrmions are topologically protected non-collinear magnetic structures. Their stability is ideally suited to carry information in, e.g., racetrack memories. The success of such a memory critically depends on the ability to stabilize and manipulate skyrmions at low magnetic fields. The non-collinear Dzyaloshinskii-Moriya interaction originating from spin-orbit coupling drives skyrmion formation. It competes with Heisenberg exchange and magnetic anisotropy favoring collinear states. Isolated skyrmions in ultra-thin films so far required magnetic fields as high as several Tesla. Here, we show that isolated skyrmions in a monolayer of Co/Ru(0001) can be stabilized down to vanishing fields. Even with the weak spin-orbit coupling of the 4d element Ru, homochiral spin spirals and isolated skyrmions were detected with spin-sensitive scanning tunneling microscopy. Density functional theory calculations explain the stability of the chiral magnetic features by the absence of magnetic anisotropy energy.

## Abstract (web summary)

Skyrmions are promising for spintronics but usually require large spin-orbit coupling of 5d-metals and external magnetic field. Here the authors realize stabilization of isolated skyrmions at a 4d-metal interface of weak chiral interaction and magnetic anisotropy down to vanishing field.

## Introduction

Topological spin structures attract rising attention due to their inherent magnetic stability^1–3^. Among these structures, isolated skyrmions are of particular interest since they can be moved by currents of very low density^4–6^. The formation of skyrmions is ultimately linked to the Dzyaloshinskii-Moriya interaction (DMI), which is a relativistic effect^7–9^ that favors non-collinear spin structures of a unique sense of rotation of neighboring magnetic moments. To display a DMI, the spatial inversion symmetry of the magnetic material needs to be broken either in the bulk by the crystal structure itself as in MnSi^10^, in layered systems by asymmetric interfaces as in [Pt/Co/Ir]_n_^11^, [Rh/Pd/2Fe/2Ir]_n_^12^, or in ultra-thin films as in Fe monolayers on a Ir(111) substrate^13^. Isolated skyrmions have been observed in a wide range of polycrystalline metallic films such as [Pt/Co/Ir]_n_^11^, CoFeB/Ta^14^ or in systems consisting of dipolar coupled magnetic films, with each of the films having non-symmetric interfaces^15,16^. However, in these polycrystalline systems, skyrmion mobility can be limited by pinning to the large amount of structural defects. For epitaxial ultra-thin films, only few systems are known to stabilize magnetic skyrmions. A monolayer (ML) of Fe/Ir(111) shows a skyrmion lattice as the ground state, but no isolated skyrmions. Only in Fe(3 ML)/Ir(111)^17^ and Pd/Fe(1 ML)/Ir(111)^18^, isolated magnetic skyrmions were reported in the presence of large magnetic fields (≈1 to 3 T).

A well established process to create skyrmions or magnetic bubble domains in thin films that display a chiral spin spiral ground state is to apply magnetic fields that cause the spirals to evolve into skyrmions^19^. The spin spiral ground state results from the competition between DMI, Heisenberg exchange, magneto-crystalline anisotropy, and dipolar energy^20–22^. As it has been shown in several theoretical works^1,2,23^, a critical DMI *D*_c_ is necessary to form spin spirals:

1 $$D_{\mathrm{c}} \propto \sqrt {Ak} ,$$

where *A* is the spin stiffness and *k* the effective magnetic anisotropy (MAE) constant. One way to stabilize the non-collinear spin structure is to enlarge the DMI above *D*_c_. This can be achieved with interfacing the magnetic atoms and 5d elements showing a large spin-orbit coupling (SOC) as it was done in Fe/Ir(111) based structures. An alternative approach taken in this work is to reduce *D*_c_ itself. *D*_c_ can be lowered to arbitrarily small values when reducing MAE to zero, rendering strong DMI an unnecessary criterion and opening up materials choice beyond 5d elements. The reduction of the MAE to zero has been used in the literature of spin-reorientation transitions (SRT)^24,25^ by adjusting the magnetic film thickness close to the compensation of magneto-crystalline anisotropy and shape anisotropy. Near the SRT, stripe domains, and magnetic bubble domains can be stabilized. The characteristic length scale of the magnetic structures are set by the magnetostatic interaction (and not the DMI)^26^. On top of this, a DMI might lead to a preferred chirality of the involved domain walls. While for thick films the long-range dipolar interaction is the dominant one and is responsible for the stabilization of stripe domains and magnetic bubbles, for ultra-thin films (in the range of 1 ML), the characteristic length scale diverges and the magnetic domains are expected to be very large.

Here, we investigate a monolayer system reported to be close to the SRT^27^: Co(1 ML)/Ru(0001). We report on the creation of isolated skyrmions in this model system. This is the first reported ultra-thin Co film system deviating from a simple collinear ground state. By using a combination of the tunneling magnetoresistance (TMR) and tunneling anisotropic magnetoresistance (TAMR) in an scanning tunneling microscopy (STM) experiment, we were able to demonstrate that both the spin-spiral and the isolated skyrmion are homochiral. In contrast to previous approaches^28,29^, this approach does not require large magnetic fields to be applied in different directions to proof a unique rotational sense. Density functional theory (DFT) calculations show that the stabilization mechanism of the isolated skyrmions differs from the ones reported in Fe/Ir(111)^13^ and Pd/Fe/Ir(111)^18^, where the DMI is the leading energy term due to large SOC. In Co/Ru(0001), the magnetic exchange interaction is stiffer by a factor of 2 and the DMI smaller by a factor of 6 as compared with Pd/Fe/Ir(111). Nevertheless, chiral magnetic states are favored due to the very weak MAE combined with a weak dipolar energy. This is the first experimental realization of this scenario initially introduced theoretically^2,23^.

## Results

### Magnetic ground state at zero magnetic field

**Fig. 1:**
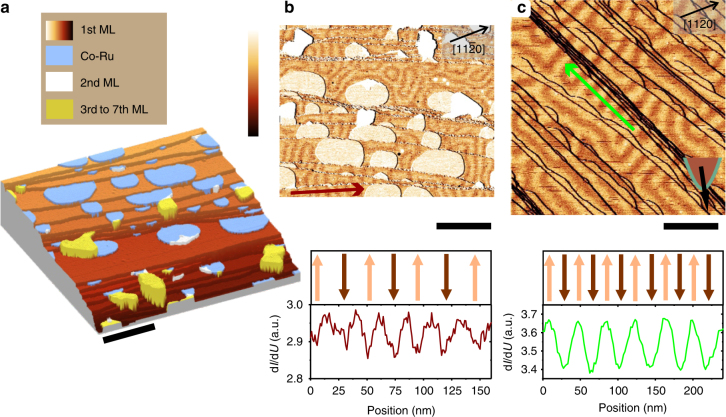
Topography and magnetic structure of Co films on Ru(0001). **a** STM topography of 1.1 ML Co (*I* = 10 nA, *U* = −320 mV, scale bar is 100 nm, height color scale goes from 0 (dark contrast) to 4.1 nm (bright contrast)). **b** Top: d*I*/d*U* map of the same area as **a** taken with a W tip (Δ*U*_rms_ = 50 mV, scale bar is 100 nm) revealing a periodic stripe pattern through TAMR effect. Bottom: line profile of d*I*/d*U* plotted along the red arrow in the image and corresponding sketch of the magnetization. **c** Top: spin polarized d*I*/d*U* map taken with a Cr coated W tip revealing the periodic magnetic stripe pattern through the TMR effect (*I* = 1 nA, *U* = −300 mV, Δ*U*_rms_ = 30 mV, scale bar is 100 nm). The tip spin polarization was out-of-plane. Bottom: Line profile of d*I*/d*U* plotted along the green arrow in the image and corresponding sketch of the magnetization

Figure 1a shows the topography of a 1.1 ML film of Co deposited on a clean Ru(0001) substrate at ≈300 °C. One ML is defined as one Co atom per substrate atom. At this deposition temperature, the first Co ML is known to grow pseudomorphically with hcp stacking forming a closed wetting layer on the substrate^30^. In agreement to this, the STM topography shows Ru atomic terraces fully covered by one Co ML. The lower edges are decorated with islands of an alloy of Co and Ru (for details, see Supplementary Fig.  1). The study of the structural and magnetic properties of these islands is beyond the scope of this communication and will be presented elsewhere. Note that the structures show a simple ferromagnetic behavior with out-of-plane magnetization. Few thicker islands (2–7 ML) are present due to the coverage being larger than 1 ML. These islands show a network of stacking faults as previously reported^30^. We performed spin-resolved STM to reveal the magnetic structure of the first ML. For this, the spatial variation of the differential tunneling conductance (d*I*/d*U*) was mapped using a bare tungsten, i.e., non-spin polarized tip (see Fig. 1b) and a spin-polarized tip (see Fig. 1c). Both maps were recorded at zero applied magnetic field. In Fig. 1b, the alloy islands, as well as the thicker islands appear brighter than the ML due to their higher density of states.

On the ML, a periodic stripe modulation is observed with a periodicity of about 20 nm (see line profile along the red arrow in Fig. 1b) when imaged using a non-magnetic tip and of about 40 nm when imaged using a spin-polarized tip with out-of plane polarization (see Fig. 1c). Since the stripe patterns in both cases react on the application of a moderate magnetic field of several 100 mT, the contrast must be of magnetic origin (see Supplementary Fig. 2). Several mechanisms may lead to stripe patterns in thin magnetic films. First of all, stripes can be stabilized by the magnetic dipolar energy. In the case of a single ML, however, the stripe periodicity is expected to diverge^31^. Second, a DMI^7–9^ or a oscillatory long-range exchange interaction^32^ may induce non-collinear ground states consisting of spin spirals. While the DMI favors a unique rotational sense of the spin spirals, i.e., a chiral spin structure^28^, the long-range exchange interaction is symmetric and spin spirals of both rotational senses are degenerate. In the spin-polarized d*I*/d*U* map (Fig. 1c), the local magnetization rotates by 180° between consecutive bright and dark stripes with the local sample magnetization pointing upward in the dark area (here upward is defines as antiparallel to the tip spin polarization) and downward in the bright area. A detailed demonstration of this is discussed in the Supplementary Fig. 2. When the spin structure is investigated with a bare W tip (Fig. 1b), the local sample magnetization can still be sensed by the TAMR^33–35^. In systems with SOC, the electronic band structure depends on the axis of the magnetization orientation giving rise to changes of the local density of states (LDOS) at specific bias voltages, i.e., areas magnetized in-plane or out-of-plane may exhibit different LDOS. Note that the TAMR contrast can also be observed in bulk-like Co and can be traced back to a TAMR in the Co surface state, as will be discussed elsewhere^36^. Thus, the experiment indicates that the local magnetization rotates by 90° between consecutive bright and dark stripes explaining the halved periodicity compared to spin-polarized measurements. In the line profiles in Fig. 1b, c, the magnetic contrast varies continuously as a function of position, i.e., the stripes are not magnetic domains separated by sharp domain walls but the magnetization gradually rotates as a function of position in the form of a spin spiral.

**Fig. 2:**
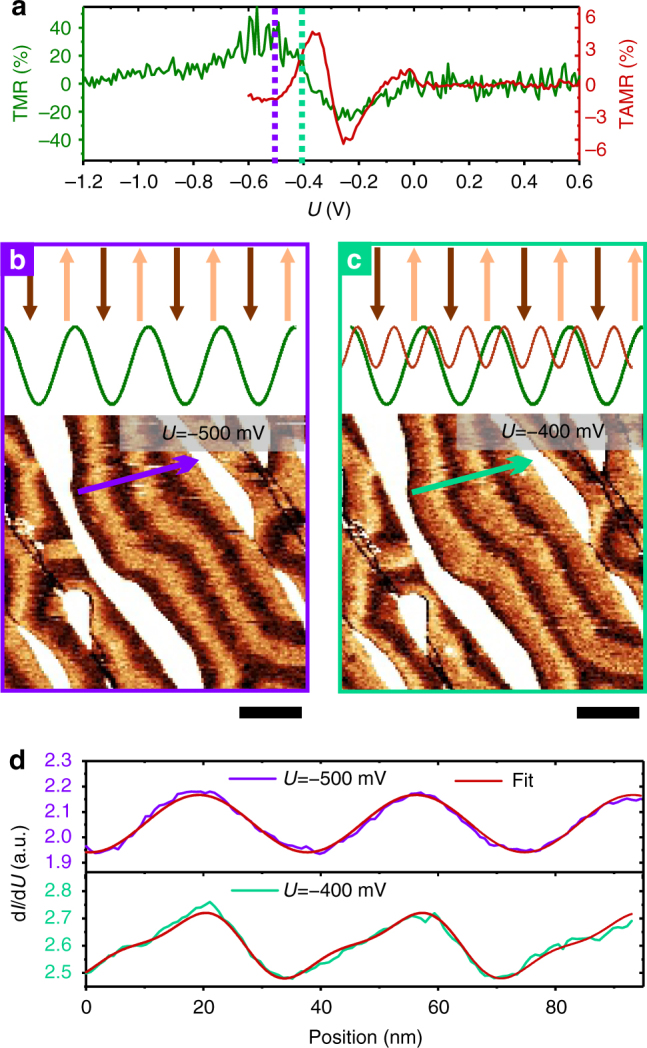
Chirality of the spin spiral. **a** Voltage dependency of the TMR (green curve—Δ*U*_rms_ = 20 mV) and TAMR (red curve—Δ*U*_rms_ = 10 mV). **b**, **c** bottom: d*I*/d*U* spin polarized map taken at *U* = −500 mV (**b**) and −400 mV (**c**) (*I* = 1 nA, Δ*U*_rms_ = 40 mV, scale bar is 50 nm, tip spin polarization was at 47° away from the normal direction). Top: sketches of the magnetization and the corresponding d*I*/d*U* profiles perpendicular to the stripes due to TMR effect (green curve) and TAMR effect (red curve). **d** Experimental d*I*/d*U* profile plotted along the violet arrow in **b** and the green arrow in **c**. Red curves superimposed on the experimental profiles are fits assuming a contribution of TMR only for the top panel and a combination between TMR and TAMR for the bottom panel

While an oscillating long-range exchange interaction will result in randomly rotating transitions, the DMI favors a specific chiral rotational sense, i.e., a homochiral spin spiral. Using in-plane polarized tips, the first will show in-plane stripes with random sequence of contrast while the latter with alternating sequence of contrast^29,37^. Further, the sign of the chirality can be achieved by a vectorial measurement of the spin polarization in large magnetic fields^38^. As the spin spirals observed in this work are strongly modified even with small fields (see Supplementary Fig. 2), we introduce an alternative method to investigate the chirality employing a combination of TAMR and TMR. The d*I*/d*U* contrast due to TAMR recorded with a bare W tip as a function of bias voltage is shown in Fig. 2a (red curve). It sensitively depends on the bias voltage, and is significant only in the range between −420 and −170 mV and displays a change of sign at −300 mV. As shown in the Supplementary Fig. 3, the TAMR at −400 mV is positive, i.e., we observe a maximal signal for an in-plane local magnetization. Respectively, the d*I*/d*U* signal is minimal when the local magnetization is out-of-plane. Note that the TAMR was determined from the contrast of ≈20 nm periodicity, i.e., between in- and out-of-plane magnetized areas. We also measured the voltage dependence of the TMR (green curve—Fig. 2a). This was determined with an out-of-plane spin-polarized tip studying the contrast with a ≈40 nm periodicity, i.e., comparing up- and down-magnetized areas. Thus, this excludes contributions of the TAMR (for more information see Supplementary Fig. 2). The TMR contrast is significant for bias voltages between −800 and −50 mV. As shown in Fig. 2a, at −500 mV (see vertical violet line in Fig. 2a), the TAMR is negligible (≈1%) in comparison to the strong TMR (≈30%). However, at −400 mV (see vertical green line in Fig. 2a), the TAMR (≈3%) and TMR (≈10%) are of comparable amplitude.

Figure 2b, c display two d*I*/d*U* maps recorded on the same area of the sample with the same spin-polarized tip at two different bias voltages: *U* = −500 mV for Fig. 2b and *U* = −400 mV for Fig. 2c. As at −500 mV, the TMR dominates, the d*I*/d*U* profile plotted along the axis perpendicular to the stripes shows a sinusoidal behavior (violet curve in Fig. 2d) that can be well fitted by a simple sine function (red curve) of 37 nm periodicity. At −400 mV, the corresponding d*I*/d*U* profile plotted in Fig. 2d (green curve) shows a “sawtooth” periodic shape. At this bias voltage, both, TAMR and TMR are present and the signal is composed of a TMR contribution (green curve, sketch top panel Fig. 2c), and a TAMR contribution (red curve, sketch top panel Fig. 2c). The TAMR signal is independent of the direction of the tip spin polarization, while the TMR signal depends on the orientation of the magnetization of the tip. The measured combination of both signals thus contains information on the direction of the tip magnetization. In order to quantitatively describe the signal, we fitted the data using the following expression:

2 $$\frac{{{\rm d}I}}{{{\rm d}U}}(x) = A_1{\rm sin}\left( {\frac{{2\pi }}{\lambda }x + \phi _1} \right) + A_2{\rm sin}\left( {\frac{{4\pi }}{\lambda }x + \phi _2} \right).$$

The first term corresponds to the TMR, the second to the TAMR signal. We fitted the experimental data using a periodicity of the spin structure of *λ* of 37 nm. Amplitudes (*A*_1_, *A*_2_) and phases (*ϕ*_1_, *ϕ*_2_) were the adjustable parameters. From the phase information, the tip spin polarization orientation was deduced to be at 47° away from the normal direction. By looking at the d*I*/d*U* map shown in Fig. 2c from left to right along the axis perpendicular to the stripes, one can see that the contrast everywhere in the image slowly rises from dark to bright, followed by a sharp transition back to dark. A non-chiral spin structure would show arbitrary changes of the rotational sense, which would lead to reversals of the sawtooth profile. As this is not the case, we conclude that a unique rotational sense is preferred in this system, i.e., the spin spiral is chiral rotates continuously by 360° per period and the DMI sets the sense of chirality. We would like to stress that the observed spiral is rotating with a constant angle between neighboring atoms, i.e., the structure is not a chiral stripe domain pattern.

In order to understand the microscopic mechanism of the spin spiral stabilization in Co/Ru(0001), we have performed DFT calculations using the FLEUR ab-initio package ((www.flapw.de (2017)); (for discussion details see Supplementary Note 4 and 5)). FLEUR uses the full linearized augmented plane wave basis set (FLAPW), which has been successful in predicting the magnetic properties of 3d monolayers hybridized with 4d or 5d non-magnetic layers^12,13,20,39^. The results of calculations for the exchange, DMI and anisotropy are summarized in Table 1.

**Table 1:** Magnetic interactions

*J*_eff_	∣D_*ij*_∣	*K*_MC_	*K*_dip_
13.1	0.2	0.015	0.07

Calculated magnetic exchange interaction, DMI, magneto-crystalline, and dipole–dipole contribution to the MAE, respectively. All coefficients are given in meV per Co atom

The exchange interaction *J*_eff_ = 13.1 meV per Co is rather large compared to other ultra-thin films hosting isolated skyrmions^12,20,21^. On the other hand, the computed DMI |**D**_*ij*_| = 0.2 meV per Co is rather small and primarily originates from the SOC of the Co layer and not from the substrate as previously reported theoretically^13,19–21,40^ and experimentally^11^ (see Supplementary Note 4). In ultra-thin films, the magneto-crystalline anisotropy is usually large and non-collinear magnetic states are not expected with such small DMI. However, several theoretical studies have concluded of a spin spiral ground state in the absence of anisotropy^1,2,23^. Our computed magneto-crystalline anisotropy value of *K*_MC_ = 0.015 meV per Co fulfills this criterion.

In order to confront our computed coefficients with experiments, we performed Monte Carlo simulation to obtain the critical temperature *T*_c_ where the ferromagnetic order vanishes. We found *T*_c_ ≈150 K in good agreement with experimental findings *T*_c_ of 170 K^27^. The magneto-crystalline anisotropy which is of the order of 15 to 50 μeV per Co atom is negligible in this system. This concord with previous experimental findings and DFT calculations^27^.

In ferromagnetic thin film of thickness close to the spin reorientation transition, the magnetic structure can be dominated by long-range dipolar interactions leading to non-homochiral spiral-like structure^41^ where the periodicity is expressed as^26^

3 $$\lambda \propto J_{{\mathrm{eff}}}a_{{\mathrm{//}}}\pi ^2{\mathrm{/}}\kappa _{\mathrm{d}},$$

where *κ*_d_ is the dipolar energy and *a*_//_ the in-plane inter-atomic distance between Co atoms. In order to verify that the dipolar energy has a negligible contribution to the stabilization of the spin spiral we have evaluated the dipole–dipole contribution to the total energy for a structural domain of size 270 × 11.7 nm^2^ (see Supplementary Note 6). The difference of energy density between in-plane and out-of-plane homogeneously magnetized domains is 72 μeV per Co and 70 μeV per Co when boundary conditions are closed and opened, respectively. In agreement with previous work^27^, we found *κ*_d_ is approximately 70 μeV per Co (see Table 1). For a spin spiral stabilized by dipolar energy, this would give a periodicity of the magnetization oscillation in the μm range, two orders of magnitude larger than the periodicity we report here. Even though the dipolar energy reinforced the spin spiral ground state, the driving force for its stabilization is the DMI and the dipolar contribution is negligible.

The spin spiral ground state we report thus results from the competition between Heisenberg exchange and DMI. Both magneto-crystalline and shape anisotropy vanish. However, as the experimental d*I*/d*U* profiles of the spiral (Fig. 2d) show, the magnetization constantly rotates and no magnetization direction is preferred further confirming a negligible MAE. In this case, the spiral period only depends on the ratio of Heisenberg exchange interaction and DMI^23^:

4 $$\lambda = 2\pi a_{{\mathrm{//}}}J_{{\mathrm{eff}}}{\mathrm{/}}D_{ij}.$$

For the Heisenberg exchange interaction given by DFT: *J*_eff_ = 13.1 meV per Co atom and the experimental periodicity *λ* = 40 nm, a *D*_*ij*_ = 0.55 meV per Co atom can be estimated. This value is in relatively good agreement with the DFT calculations (see Supplementary Note 4).

The ground state of the ML of Co/Ru(0001) is, therefore, a chiral spin spiral degenerate with the ferromagnetic state. For this ultra-thin film close to the spin reorientation transition, magnetic anisotropy as well as dipolar interaction are negligible. Even though only light elements (Co: 3d, Ru: 4d) exhibiting weak SOC are used in this structure, the small DMI stabilizes a chiral spin spiral.

### Magnetic skyrmions

**Fig. 3:**
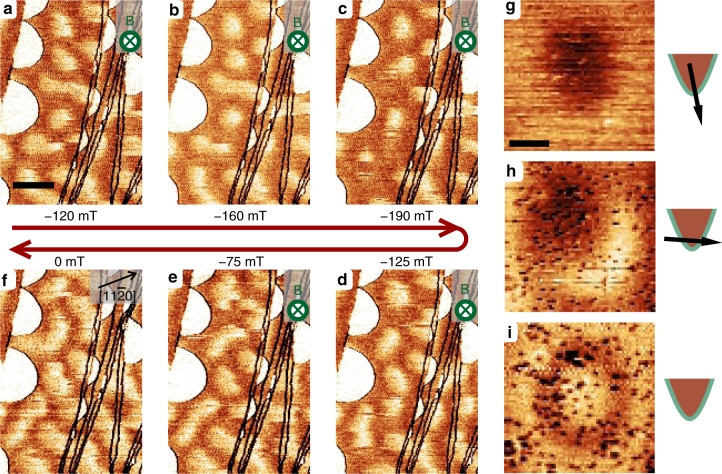
Stabilization of magnetic skyrmions. **a**–**f** Spin-polarized d*I*/d*U* maps of the same area taken with out-of-plane magnetic fields as indicated (*I* = 1 nA, *U* = −400 mV, Δ*U*_rms_ = 40 mV, scale bar is 50 nm). **g**–**i** Magnified d*I*/d*U* maps recorded on the same skyrmion with an out-of-plane (**g**), in-plane (**h**), and non (**i**) spin-polarized tip (*I* = 1 nA, *U* = −400 mV for **g** and **h** and −200 mV for **i**, Δ*U*_rms_ = 40 mV for **g** and **h** and 30 mV for **i**, scale bar is 10 nm)

Figures 3a–f display six spin-polarized d*I*/d*U* maps recorded consecutively with an out-of-plane spin-polarized tip at perpendicular magnetic fields as indicated. As already discussed above and in the Supplementary Fig. 2, the magnetic structure is significantly modified even by modest fields. Dark areas expand with the applied out-of-plane field indicating a local orientation parallel to that of the field. Some of the remaining bright areas where the magnetization is antiparallel to the field tend to form circular dots of anti-aligned magnetization in an environment of magnetization aligned with the field. Upon further increase of the magnetic field (Figs 3a–c) some of these dots disappear when encountering structural defects such as step edges. When decreasing the magnetic field from −190 to 0 mT (Figs 3c–f), the dots elongate to worm-like domains and finally to the spin-spiral structure. This behavior has is analogous to the elliptical instability of bubble domains^1,22^. After ramping the field down to zero, some circular dots remained stable. This behavior is mostly found on narrow atomic terraces, as is discussed in the Supplementary Note 7. Figures 3g–i show three d*I*/d*U* maps of such circular spin structures. As for Cr coated W tips, the spin polarization is extremely sensitive to the tip termination, slight voltage pulses were sufficient to switch the tip spin polarization from out-of-plane (Fig. 3g) to in-plane (Fig. 3h) to unpolarized (Fig. 3i).

First, we focus on the results obtained with an unpolarized tip. Figure 3i was recorded at −200 mV, where the TAMR is negative, i.e., in-plane magnetization give a lower d*I*/d*U* signal. The map clearly shows a continuous transition of an out-of-plane magnetization in the center of the spin structure via an in-plane orientation (dark ring) back to an out-of-plane orientation with a similar rate as observed in the chiral spin spiral. Thus, the structure does not represent a bubble domain with sharp domain walls but resembles a proper skyrmion. Figure 3g, h give information on the direction of rotation of the magnetization. The out-of-plane image indicates that the center of the skyrmion is magnetized in opposite direction of the surroundings and the in-plane image reveals a specific rotation sense of the spin structure. The contrast reveals two lobes: the bright and dark one correspond to a tip spin polarization parallel and antiparallel to the local sample magnetization, respectively. Note that all observed circular spin structures display the same orientation of the in-plane contrast confirming the role of DMI (see Supplementary Note 7). Further note that the two images were recorded at −400 mV, where also a small TAMR is present leading to a slight overshoot of the d*I*/d*U* signal in Fig. 3g. We can conclude that the topology of the observed spin structure is that of skyrmions, i.e., it exhibits a winding number of 1. This observation is in agreement with the unique chirality of the spin spiral discussed above.

As a result of the small energy difference between the spin spiral and ferromagnetic state (i.e., low magnetic anisotropy and low DMI) in this system, isolated skyrmions can be created at rather low magnetic field (≈150 mT) and are (meta)stable in the remanent state. This is in sharp contrast to other epitaxial ultra-thin film model systems, where large magnetic fields are needed and skyrmions are absent in the remanent state^18^.

In order to study skyrmion stability with respect to the spin spiral and the ferromagnetic states, we minimized their total energy via spin dynamics simulation. We solved the LLG equation using the extended Heisenberg model parametrized from our DFT calculation (see Supplementary Note 8). Under a magnetic field of 150 mT, the ferromagnetic state with a magnetization aligned to the field, the spin spiral state and the skyrmion state are quasi-degenerate. For higher magnetic field, the skyrmions are metastable with respect to the ferromagnetic state in agreement with the experimental observation.

**Fig. 4:**
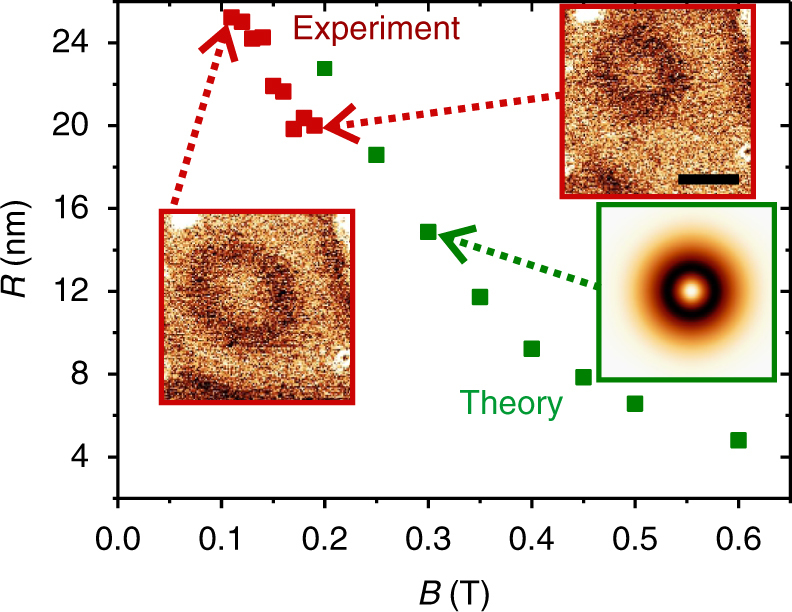
Magnetic field dependency of skyrmion radius. Dependency of the skyrmion radius with the magnetic field—Green dots correspond to to radius extracted from Monte Carlo simulations—Red dots are extracted from experiments realized with a bare tungsten tip—inset red frame: two example of the experimental d*I*/d*U* map used to extract this profile showing a single skyrmion through the TAMR effect—the left one was recorded at 120 mT—the right one at 190 mT (60 × 60 nm, *I* = 1 nA, *U* = −220 mV, Δ*U*_rms_ = 50 mV)—inset green frame: Theoretical d*I*/d*U* TAMR contrast of an isolated skyrmion at 300 mT, scale bar is 20 nm

Finally, we have studied the dependence of the skyrmion size on a small perpendicular magnetic field. As the skyrmions can be stabilized at rather low fields, any stray field of the tip would modify the skyrmion structure or laterally move it during scanning (see Supplementary Note 7). To avoid this, a bare tungsten tip was used to image the skyrmion with the TAMR at −220 mV. We varied the perpendicular field within the experimentally accessible range between 110 and 190 mT in the direction antiparallel to the core of the skyrmion, i.e., the field is expected to compress the skyrmion. Two of the d*I*/d*U* maps recorded are displayed in the inset of Fig. 4. From 120 to 190 mT, the diameter of the dark ring, i.e., the in-plane oriented section, is reduced by about 5 nm. Here, the radius was determined by fitting the d*I*/d*U* data to a two-dimensional radial cosine function^11^ (see Supplementary Note 9). Figure 4 also contains the skyrmion radius obtained from atomistic spin dynamics simulations parametrized by our DFT calculation (see Supplementary Note 8 and method section). For magnetic fields above 200 mT, skyrmions show stable radii. The skyrmion radii obtained from experiments (red points) and numerical simulations (green points) agree well within few nm precision. This further confirms that the parameters obtained from DFT calculations (magneto-crystalline anisotropy, DMI, Heisenberg exchange, and dipolar energies) are able to model the experimental findings. Note that annihilation of isolated skyrmions is hampered due to their topological charge. In order to destroy this charge without interaction of the skyrmion with an island edge, a magnetic singularity needs to be injected into the spin structure. This mainly needs exchange energy and thus, the large exchange observed in the Co films is responsible for the (meta)stability of the skyrmions^42,43^.

## Discussion

In conclusion, we demonstrate that skyrmions can be formed at low magnetic fields without the requirements of large spin orbit coupling. Then, skyrmions remain (meta)stable in the remanent state. The stability of isolated skyrmions in Co/Ru(0001) in combination with their high mobility (see Supplementary Note 7) makes Co/Ru(0001) an ideal model system to study skyrmion manipulation and skyrmion dynamics. Furthermore, the experimental discovery of isolated skyrmions with a vanishing MAE in combination with a low DMI at 4d-metal interfaces can be used as a general direction to obtain skyrmions in 3d–4d thin-films suitable for lithography and application. To the best of our knowledge, Co/Ru(0001) is the first system of that kind and has to distinguished from stripe or bubble domains with chiral walls. In our case, the ground state at vanishing fields are continuously rotating spin spirals and skyrmions are not of a bubble domain type. Ultimately, we report the first evidence of the presence of isolated skyrmions on a possible superconductor. Ru becomes superconducting below 500 mK and the formation of Majorana states attached to magnetic skyrmions at the interface are expected. Isolated skyrmions in Co/Ru(0001) offers the perspective to use this system for topological quantum computing^44–46^.

## Methods

### Experiments

Samples and tips were prepared under ultra-high vacuum (UHV) at a base pressure of 4 × 10^−11^ mbar. Unpolarized STM tips were prepared from a W wire and were cleaned in-situ by flashing above 2800 °C. Spin-polarized tips were prepared by depositing a Cr thin film onto the tip followed by a gentle annealing^47^. The Ru(0001) single crystal was cleaned by cycles of annealing in oxygen at 1000 °C followed by flashing to 1500 °C. Once the substrate was depleted from bulk carbon impurities, cycles of argon-ion sputtering and annealing to 1500 °C were performed to obtain atomically flat and clean surfaces. The Co film was deposited from an e-beam evaporator onto the clean Ru surface with a deposition rate of 0.3 ML per minute. Tips and samples were directly transferred to the STM under UHV. STM measurements were performed at 4.2 K with a home-built microscope.

### Density functional theory

For the DFT study, the FLAPW basis as implemented in the FLEUR ab-initio package was used, which accurately describes the ultra-thin film geometry by considering different basis function for the vacuum, the atomic muffin tin (MT) and the interstitial region. Within this framework, we have relaxed a symmetric Co/Ru(0001) slab composed of two Co monolayers separated by five layers of Ru. We have used a mixed LDA/GGA exchange and correlation functional that treats the MT of Ru in LDA and the MT of Co in GGA. In detail, we have used the Vosko LDA^48^ and the GGA-PBE^49^. In order to ensure a good convergence, we have used a cutoff of the plane waves basis set (*K*_max_) of 4.0 bohr^−1^ and 110 k−points in 1/12^th^ of the first Brillouin zone (BZ) of the hexagonal unit cell. The muffin tin radius of Co and Ru are 2.27 and 2.4 bohr, respectively. After the structural relaxation was performed, the spin-spiral energies were calculated in a unit cell containing one Co atom and 5 Ru atoms. The dispersion curves were calculated via the generalized Bloch theorem. The SOC contribution was calculated via first order perturbation theory^50^. The magnetic force theorem^51^ was used to calculate the 54 and the 27 nm spin spiral with and without SOC contribution. We have used a 300 × 300 k-points mesh and *K*_max_ was set to 4.3 bohr^−1^ for both calculations. We have calculated the MAE as the energy difference between several magnetic configurations in a supercell containing one Co on nine Ru layers. We have considered three different cases: An out-of-plane easy axis, an in-plane easy axis along the $\it \bar \Gamma - \bar K$ and along the $\it \bar \Gamma - \bar M$ direction. All magnetic configurations were converged self-consistently. We have used *K*_max_ = 4.3 bohr^−1^, 44 × 44, 63 × 63, and a 83 × 83 k-points mesh in the full BZ. The calculations give a MAE of 15 μeV per Co, 40 μeV per Co, and 50 μeV per Co, respectively. We have used 15 μeV per Co for the Monte Carlo and the spin dynamics simulations. Higher values of MAE would not change the qualitative agreement between theory and experiments.

### Spin dynamics simulations

We have mapped our DFT calculations on the magnetic Hamiltonian:

5 $$\begin{array}{ccccc} H = - \mathop {\sum}\limits_{ij} J_{{\mathrm{eff}}}{\mathbf{M}}_i \cdot {\mathbf{M}}_j - \mathop {\sum}\limits_{ij} {\mathbf{D}}_{ij} \cdot \left( {{\mathbf{M}}_i \times {\mathbf{M}}_j} \right)\\ + \mathop {\sum}\limits_i K\left( {{\bf M}_i^z} \right)^2 + {\mu}_{0}{\mu}_{\rm B} M_{\rm s} \mathop {\sum}\limits_i {\mathbf{B}} \cdot {\mathbf{M}}_i \\ \end{array}$$

where *J*_eff_ is the magnetic exchange interaction close to the FM ground state, **D**_*ij*_ is the Dzyaloshinskii-Moriya interaction, *K* is the uniaxial anisotropy vector and **B** is the external magnetic field. The profile and energy stability of the isolated skyrmions were relaxed via spin dynamics using the Landau-Lifschitz-Gilbert equation (LLG):

6 $$\hbar \frac{{{\rm d}{\mathbf{M}}}}{{{\rm d}t}} = {\mathbf{M}} \times {\mathbf{B}}_{{\mathrm{eff}}} - \alpha {\mathbf{M}} \times \left( {{\mathbf{M}} \times {\mathbf{B}}_{{\mathrm{eff}}}} \right),$$

where *ħ* is the Planck constant and **B**_eff_ = −d*H*/d**M** is the effective field created by the neighboring magnetic moments. We have integrated the LLG equations with a Heun integrator. The maximal torque $\left\| {\mu _0{\mathbf{B}}_{{\mathrm{eff}}} \times {\mathbf{M}}} \right\|$ was converge down to 2.10^−6^ eV.

### Data availability

All the data are available from the authors upon reasonable requests.

## Electronic supplementary material

Supplementary Information
