# Publisher Correction: Stabilizing spin spirals and isolated skyrmions at low magnetic field exploiting vanishing magnetic anisotropy

**DOI:** 10.1038/s41467-018-04680-0

**Published:** 2018-06-01

**Authors:** Marie Hervé, Bertrand Dupé, Rafael Lopes, Marie Böttcher, Maximiliano D. Martins, Timofey Balashov, Lukas Gerhard, Jairo Sinova, Wulf Wulfhekel

**Affiliations:** 1 Physikalisches Institut, Karlsruhe Institute of Technology, 76131 Karlsruhe, Germany; 2 Institut für Physik, Johannes Gutenberg Universität Mainz, 55099 Mainz, Germany; 3 Institute of Theoretical Physics and Astrophysics, University of Kiel, 24098 Kiel, Germany; 4 Centro de Desenvolvimento da Tecnologia Nuclear, 31270-901 Belo Horizonte, Brazil; 5 Institute of Nanotechnology, Karlsruhe Institute of Technology, 76128 Karlsruhe, Germany; 6 Institute of Physics, Academy of Sciences of the Czech Republic, Cukrovarnická 10, 162 53 Praha 6, Czech Republic

Correction to: *Nature Communications*, 10.1038/s41467-018-03240-w, published online 09 March 2018

The original version of this Article had an incorrect Received date of 21 November 2016; it should have been 21 November 2017. This has been corrected in the PDF and HTML versions of the Article.

