# Supplementary material for: Stabilizing spin spirals and isolated skyrmions at low magnetic field exploiting vanishing magnetic anisotropy
Source: Nat Commun. 2018 Mar 9;9:1015. doi: 10.1038/s41467-018-03240-w (PMC5844874; doi:10.1038/s41467-018-03240-w)
Supplement: Supplementary file 1 — Supplementary Information [file 41467_2018_3240_MOESM1_ESM.pdf]

Supplementary Note 1. Interdiffusion between Co and Ru

As mentioned in previous studies [1], interdiffusion between Co and Ru occurs at deposition temperatures above 250 °C. The STM topography Fig. 1a in the main text shows that for 1.1 Co ML deposited at ≈ 300 °C. the lower step edges of Ru are decorated by islands appearing brighter (higher dI/dU) than the Co wetting layer in the dI/dU map. Additional investigations of the density of these islands for different deposition temperatures are displayed in supplementary fig. 1. 1.1 Co ML was deposited at 250 °C (supplementary fig. 1a) and 350 °C (supplementary fig. 1b). Increasing the deposition temperature from 250 °C to 350 °C leads to an increase of the density of these islands. We interpreted the nature of these islands as an alloying between Co and Ru. Detailed informations will be presented elsewhere.

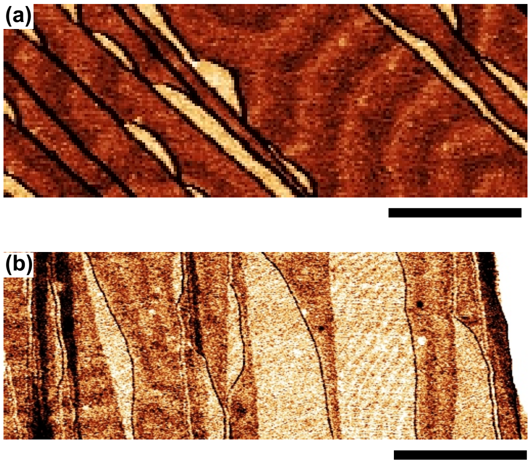

Supplementary Figure 1. **Alloy islands.** dI/dU maps of 1.1 Co ML deposited at 250 °C (a) and 350 °C (b). The bright islands decorating the lower step edges are ascribed to an alloy of Co and Ru. The density of these islands increased with deposition temperature (I=1 nA, ΔU_{rms}=40 mV, (a) U=−400 mV, (b) U=−290 mV, scale bar is 100 nm). The pure Co areas show the spin spiral contrast probed with a spin-polarized tip in (a) and a non-spin polarized tip in (b).

Supplementary Note 2. Magnetic field dependency of the spin polarized contrast

The periodic stripe pattern shown in Fig. 1c in the main text can be modified by the application of an out-of-plane magnetic field. Moderate positive magnetic fields (B=+190 mT, cf. supplementary fig. 2b) lead to an expansion of the bright areas, while negative magnetic

fields (B=−190 mT), cf. supplementary fig. 2d) lead to the expansion of the dark areas. Decreasing B from +190 mT to 0 mT regenerates the balance between dark and bright areas (cf. supplementary fig. 2c). This indicates that the observed bright and dark contrast areas are due to a local out-of-plane orientation of magnetization. Note that for the small field used here, the spin-polarization direction of the tip is not affected [2].

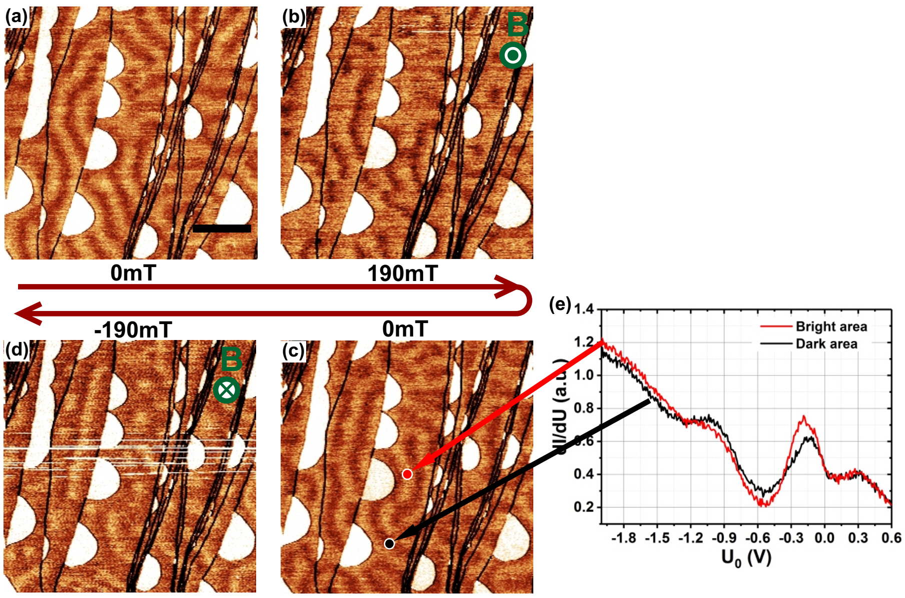

Supplementary Figure 2. **Magnetic field dependency of the stripe pattern.** (a) - (d) dI/dU maps of the same area recorded consecutively with an out-of-plane spin-polarized tip at an out-of-plane magnetic field as indicated (I=1 nA, U=−500 mV, ΔU_{rms}=40 mV, scale bar is 100 nm). (e) dI/dU spectra recorded in the middle of a bright stripe where the local magnetization is parallel to the tip spin polarization (red curve) and in the middle of a dark stripe where the local magnetization is antiparallel to the tip spin polarization (dark curve) (I=1 nA, U=−1.5 V, ΔU_{rms}=20 mV).

The voltage dependency of the TMR effect presented in Fig. 2a in the main text was obtained from the two curves presented in supplementary fig. 2e. dI/dU spectra were recorded by stabilizing the tip at -1.5 V, disabling the feedback and ramping the voltage with the tip located in the middle of a bright stripe (red curve) and in the middle of a dark stripe (dark curve). The relative variation of the dI/dU signal between the spectra measured on the dark and the bright stripe was converted to the voltage dependency of the

TMR (Fig. 2a - main text) as:

$$TMR = \frac{\frac{dI}{dU}_{\text{dark}} - \frac{dI}{dU}_{\text{bright}}}{\frac{dI}{dU}_{\text{dark}}}. \quad (1)$$

Supplementary Note 3. Magnetic field dependency of the TAMR contrast

The evolution of the periodic stripe pattern under an out-of-plane magnetic field was recorded with non-spin polarized tip as well. supplementary fig. 3a shows a dI/dU map recorded at a bias voltage of -250 mV on an isolated Co island. The darkest zone in this image corresponds to the bare Ru substrate. The contrast inside the island shows the periodic stripe pattern. supplementary fig.3a to h display half of a magnetization cycle in this isolated Co island. For an out-of-plane magnetic field of -125 mT (supplementary fig. 3c) and $+150$ mT (supplementary fig. 3h), the magnetization is almost saturated and is aligned parallel with the magnetic field. For both positive and negative magnetic field, an expansion of the bright area is observed. The maximum of the dI/dU signal (bright contrast) corresponds to an out-of-plane magnetization direction (either up or down). An in-plane magnetization gives a minimum of the dI/dU signal (dark contrast). The observed contrast is interpreted as originating from an out-of-plane TAMR effect where the dI/dU varies as a cos^2 with the angle formed between the magnetization and an out-of-plane quantization axis [3–5]. Note that in the saturated magnetic state (supplementary fig. 3c and h), dark contrast patches still remain on the island edges. At these locations, the magnetization is twisted. While decreasing the magnetic field toward 0 mT (supplementary fig. 3d and e), the spin spiral state is regenerated.

The voltage dependency of the TAMR effect presented in Fig. 2a in the main text was obtained from the two curves presented in supplementary fig. 3i. dI/dU spectra were recorded with the tip located in the middle of a bright stripe (red curve - out-of-plane magnetization) and in the middle of a dark stripe (dark curve - in-plane magnetization). The relative variation of the dI/dU signal is converted to the voltage dependency of the TAMR effect (Fig. 2a - main text) as:

$$TAMR = \frac{\frac{dI}{dU}_{\text{in-plane}} - \frac{dI}{dU}_{\text{out-of-plane}}}{\frac{dI}{dU}_{\text{in-plane}}}. \quad (2)$$

As explained in the main text, an inversion of the TAMR occurs at -300 mV. supplementary fig. 3 shows the dI/dU maps at a bias voltage of -250 mV. In that case, the TAMR

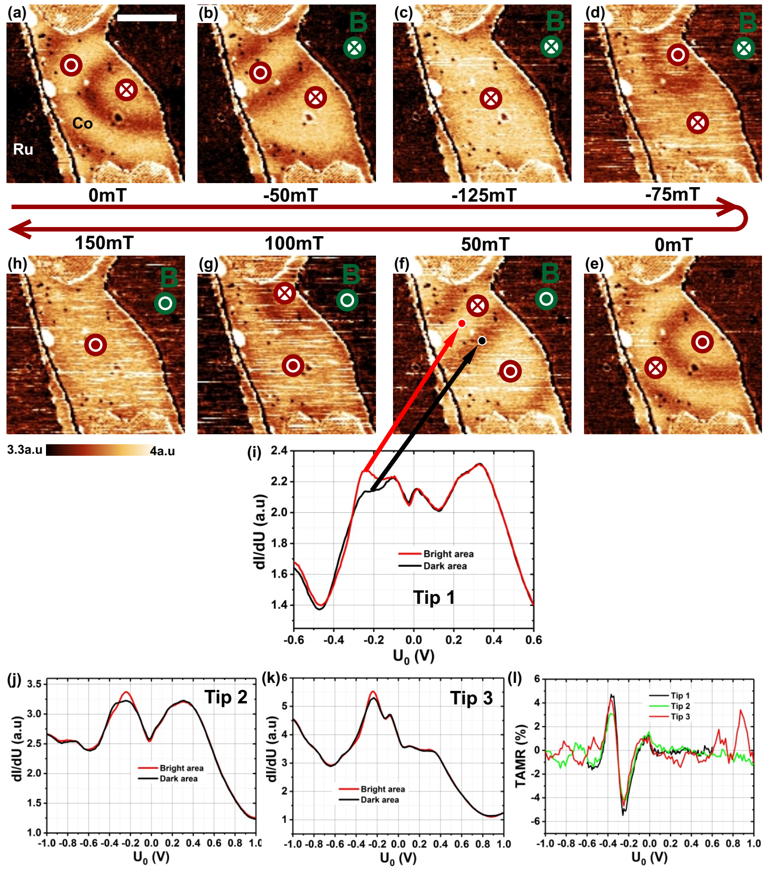

Supplementary Figure 3. **Magnetic field dependency of the TAMR contrast.** (a) - (h) dI/dU maps recorded at out-of-plane magnetic fields as indicated. The tip was unpolarized and the spin spiral was probed through TAMR (I=1 nA, U=−250 mV, ΔU_{rms}=30 mV, scale bar is 25 nm). (i) - (k) dI/dU spectra recorded on a bright stripe (red curve - local magnetization out-of-plane) and on a dark stripe (dark curve and local magnetization in-plane) with three different tips ((i): I=10 nA, U=600 mV, ΔU_{rms}=10 mV - (j) and (k): I=10 nA, U=1000 mV, ΔU_{rms}=20 mV). (l) Voltage dependency of the TAMR for the three different tips.

is negative, a maximum of the dI/dU signal corresponds to an out-of-plane magnetization, a minimum of the dI/dU amplitude coincide with an in-plane magnetization. In contrast, Fig. 2c (main text) shows the dI/dU map recorded at a bias voltage of −400 mV. Then, the TAMR is positive and the magnetization pointing out-of-plane correspond to a minimum of the dI/dU amplitude, while in-plane magnetization shows a maximum of the dI/dU signal.

Note that in scanning tunneling spectroscopy measurements, sample and tip LDOS are convoluted. By means of voltage pulses it was possible to change the tip termination and its

LDOS. supplementary fig. 3j and k display two other kind of dI/dU spectra recorded with two other tip termination. The general shape of the dI/dU spectra is strongly affected by the tip LDOS. However, the TAMR feature (see supplementary fig. 3l) remains unchanged.

Supplementary Note 4. Evaluation of magnetic interaction parameters

The magnetic interactions, were calculated by exploring total energy of spin-spirals [6] characterized by a constant angle between neighboring magnetic moments $\theta_i = \mathbf{q} \cdot \mathbf{r}_i$. Here $\mathbf{q}$ is the propagation direction of the spin spiral and $\mathbf{r}_i$ is the position of the ith magnetic moment. In the case of Co/Ru(0001), the experimental angle between neighboring spins is rather small ($\theta = 2.4°$) which correspond to a propagation vector of $q = 0.0067\, 2\pi/a$. This justified to consider the spin spiral as a perturbation from the ferromagnetic (FM) state, i.e. to use the magnetic force theorem [7, 8].

supplementary fig. 4(a) shows the energy of the spin spiral with respect to the FM state with and without SOC for small q. As the angle between adjacent spins increases, the exchange energy rises quadratically. Without SOC, both right- and left-rotating states are degenerate (blue curve) and the exchange interaction alone favors a FM state ($q = 0$). When SOC is taken into account, the degeneracy is lifted and a metastable energy minimum is created for the left rotating spin spiral at $q = 0.0025\ 2\pi/a$ (red curve). This metastable state is degenerate only 1.4 μeV per Co above the FM state.

To disentangle the different magnetic interactions (Heisenberg exchange and DMI), we have mapped our DFT result onto a Heisenberg Hamiltonian.

$$\mathcal{H} = -\sum_{i,j} J_{\text{eff}} \left(\mathbf{M}_i \cdot \mathbf{M}_j\right) - \sum_{i,j} \mathbf{D}_{ij} \cdot \left(\mathbf{M}_i \times \mathbf{M}_j\right), \qquad (3)$$

Where J_{eff} is the magnetic exchange interaction, $\mathbf{D}_{ij}$ is the DMI and $\mathbf{M}_j$ are unit vectors collinear to the magnetic moments at position $\mathbf{r}_i$ and $\mathbf{r}_j$, respectively. The Heisenberg exchange contribution J_{eff} was determined from a fit to the dispersion curve $E(q)$ of the spin spiral when SOC is not included. The value can be approximated to the first neighbor exchange interaction and higher order term are negligible here. We found $J_{\text{eff}} = 13.1$ meV/Co.

We obtained the DMI by fitting the SOC contribution to $E(q)$ shown in supplementary fig. 4(b). We use the DMI model of Levy and Fert [9], which approximates $\mathbf{D}_{ij}$ as:

$$\mathbf{D}_{ij} = V\left(\lambda_D\right)\left(\mathbf{r}_i \times \mathbf{r}_j\right), \qquad (4)$$

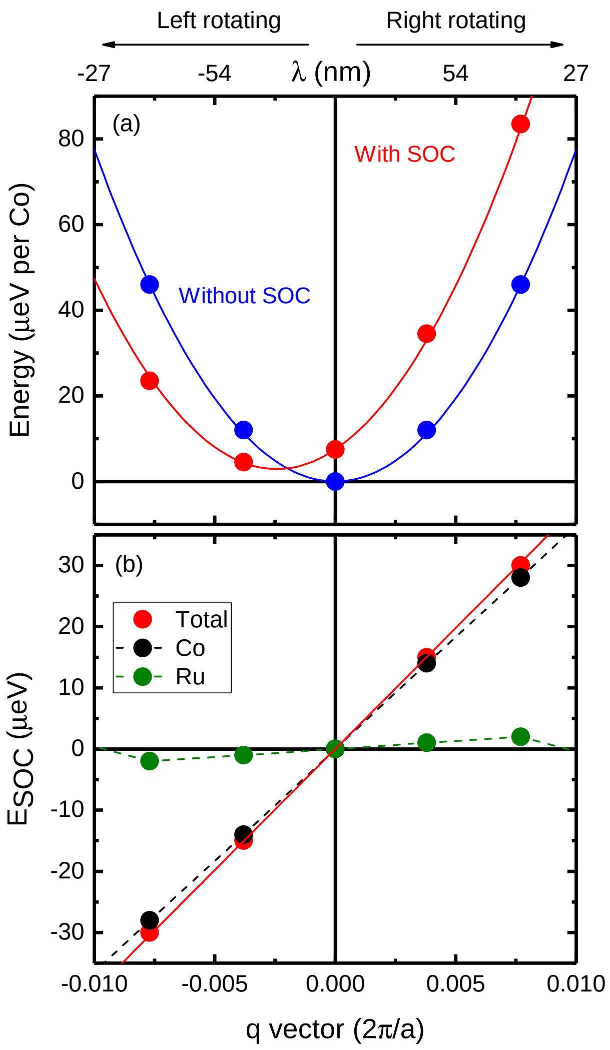

Supplementary Figure 4. **Spin spiral dispersion curve.** (a) Energy of a left and right rotating flat spin spiral as a function of the **q** vector (in cartesian coordinate) along the $\bar{\Gamma} - \bar{\mathrm{M}}$ direction. The continuous lines correspond to fits of the dispersions curve close to the $\bar{\Gamma}$-point ($\mathbf{q} = 0$) without SOC (blue) and with SOC contribution (red). (b) Layer decomposition of the SOC contribution to the dispersion curve. The continuous line correspond to a fit of the SOC contribution on the effective DM interaction written in eq. 3. On both panels, the dashed magenta and orange lines correspond the dispersion curve with SOC contribution when the DMI is set to $D_1 = 0.3$ meV and $D_1 = 0.4$, respectively.

where $\mathbf{r}_i$ and $\mathbf{r}_j$ are the position of two magnetic atoms (in Co) with respect to a non-magnetic atom (Ru in our case) and λ_D is the strength of SOC of the d-electrons of the non-magnetic

atom on the conduction electrons and $V(\lambda_D)$ is a perturbing potential depending linearly on λ_D (for more details see [9]). supplementary fig. 4b shows the total contribution (red line), the Co contribution (black line) and the Ru contribution (green line) to the SOC. In the case of Co/Ru(0001), the DMI is induced by the SOC of Co and not of the Ru substrate in contrast to previous works. In that respect, Co/Ru(0001) is the first ultra-thin film where the spin spiral ground state is induced by the SOC of the magnetic monolayer. We have obtained $\mid \mathbf{D}_{ij} \mid = -0.2$ meV/Co. The minus sign corresponds to the stabilization of a left rotating spin spiral.

Supplementary Note 5. Estimation of $\mathbf{T}_{\mathrm{c}}$

In order to compare the coefficients of our effective atomistic spin model with experiments, we performed Monte Carlo simulation to determine the critical temperature T_{c} at B=0 T. We have used a standard metropolis Monte Carlo method. To decrease the computational costs, we modeled a slab of Co/Ru(0001) using an asymmetric supercell composed of 200×10 magnetic moments along the $\bar{\Gamma} - \bar{M}$ direction. We have used $J_{\mathrm{eff}} = 13.1$ meV/Co, $D_{ij} = -0.2$ meV/Co and $K = 0.015$ meV/Co. The starting configuration is a ferromagnetic state where the magnetic moments are pointing along the long axis of the slab. We have computed 100 equally spaced temperatures within the range [1 K, 650 K]. For each temperature, the magnetic moments were first thermalized 2×10^5 MC steps. The energy, magnetization densities and the magnetic susceptibility were averaged over 5×10^6 MC steps, each of them separated by 2×10^4 autocorrelation steps.

In order to estimate the Curie temperature, we analyse the energy and the magnetization density. We compute the specific heat as:

$$C = \frac{\langle E^2 \rangle - \langle E \rangle^2}{k_{\mathrm{B}} T^2}, \tag{5}$$

where $\langle E \rangle$ is the average energy density, k_{B} is the Boltzmann constant and T is the temperature. We have computed the magnetic susceptibility as:

$$\chi_M = \frac{\langle M^2 \rangle - \langle M \rangle^2}{k_{\mathrm{B}} T}. \tag{6}$$

We have modeled the energy and the magnetization density as arctangent function. We can then fit C and χ_M with the Lorentzian function:

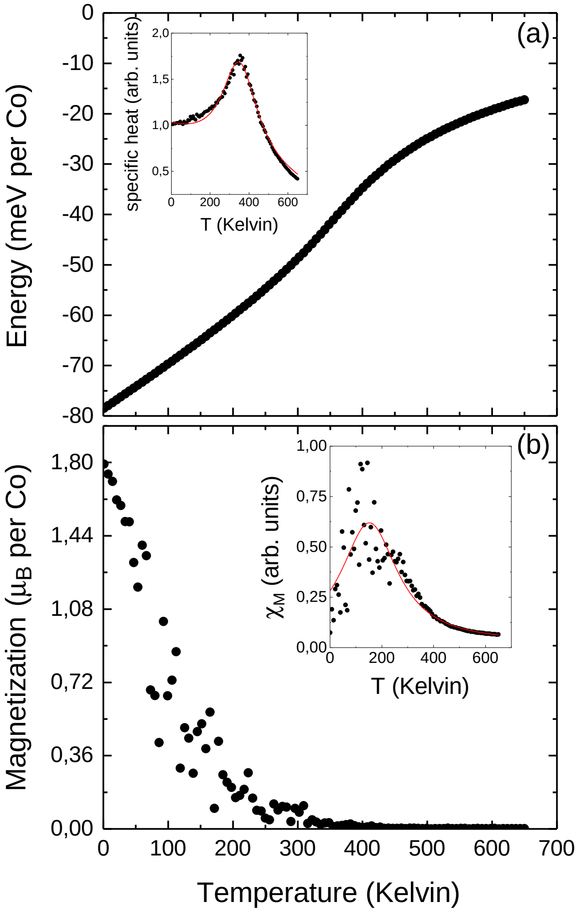

Supplementary Figure 5. **Determination of T_c.** (a) Energy density as a function of temperature. The inset shows the specific heat as a function of temperature. The peak of specific heat corresponds to the phase transition between the ordered and disordered phases. (b) Magnetization density as a function of temperature. The magnetization disappears at $T \sim 200$ K. The inset shows the magnetic susceptibility and a lorentzian fit (red line). We extract from the fit $T_c^M = 152$ K.

$$F(T) = F_0 + \frac{2I}{\pi} \frac{\sigma}{4\left(T - T_c\right)^2 + \sigma^2}, \tag{7}$$

where I is the intensity of the peak and σ is the mean-height width. All the fits were carried out with a linear background. By fitting of the specific heat, we obtain $T_c = 350$ K. This critical temperature corresponds to the transition between the ordered phase to the

disordered phase. Ref. [10] reports on the temperature at which the magnetization vanishes i.e. $T_c^M = 170$ K. When the susceptibility χ_M is fitted with the Lorentzian curve, we found $T_c^M = 152$ K, in good agreement with the experimental value.

Supplementary Note 6: Dipole-dipole interaction in Co/Ru(0001)

We have estimated the strength of the dipole-dipole interaction. The dipole-dipole energy density is given by:

$$E = \frac{\mu_0 \mu_s^2}{4N\pi} \sum_{i,j=1}^{N} \frac{(\mathbf{m}_i \cdot \mathbf{m}_j)\, r_{ij}^2 - 3\,(\mathbf{m}_i \cdot \mathbf{r}_{ij})\,(\mathbf{m}_j \cdot \mathbf{r}_{ij})}{r_{ij}^5}, \tag{8}$$

where $N = \sqrt{3}S/2a^2$ is the number of magnetic moments, S is the surface and a is the lattice parameter ($a = 0.27$ nm), μ_0 is the magnetic permeability of vacuum ($\mu_0 = 5.788.10^{-5}$ eV/T), μ_s is the magnetic moments of Co ($\mu_s = 1.8\ \mu_B$), $\mathbf{m}_i$ the magnetization unit vector at position $\mathbf{r}_i$ and $\mathbf{r}_{ij} = \mathbf{r}_j - \mathbf{r}_i$. To quantify the energy density originating from the dipole-dipole interaction, we compare the situation where the magnetization points in different direction for a hexagonal domain of size 270×11.7 nm^2 with closed and open boundary conditions.

We start by calculating the total energy of the spin-spiral which was obtained from our DFT calculations. When the dipole-dipole energy is included, this spin spiral configuration is the ground state. If we consider homogeneously magnetized states, as expected, the out-of-plane magnetized domain is the maximum of energy. The dipole-dipole energy difference between the out-of-planed and the in-planed magnetized domain is $70\,\mu$eV/Co and $72\,\mu$eV/Co with open and closed boundary conditions, respectively and favors an in-plane magnetization .

Supplementary Note 7. Metastable skyrmions at zero magnetic field and skyrmion mobility

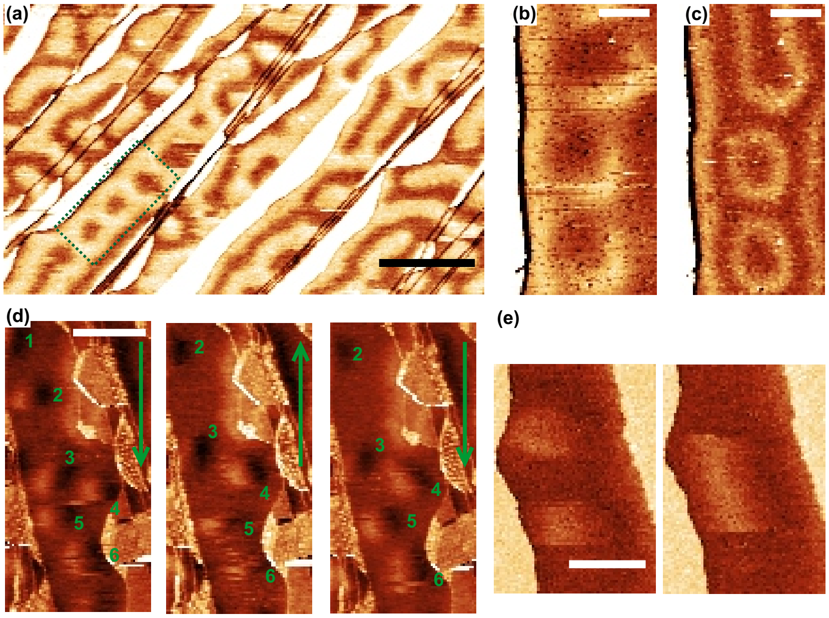

Supplementary Figure 6. **Metastable skyrmions at zero magnetic field and skyrmion mobility.** (a) to (c) dI/dU maps showing remaining skyrmions after ramping down the magnetic field from −150 mT to 0 mT. (a) The tip was out-of-plane spin-polarized (I=1 nA, U=−400 mV, $\Delta U_{\rm rms}$=40 mV, scale bar is 100 nm). (b) and (c) were recorded in the area marked in (a) after two consecutive tip changes. The tip was in-plane spin-polarized in (b) and unpolarized in (c) (I=10 nA, U=−400 mV,$\Delta U_{\rm rms}$=40 mV, scale bar is 20 nm). (d) Three dI/dU maps recorded consecutively with an in-plane spin-polarized tip showing 6 magnetic skyrmions under a magnetic field of 150 mT and showing than skyrmions are moved while the tip was scanned (scale bar is 50 nm). (e) Two dI/dU maps realized consecutively with an out-of-plane spin-polarized tip showing the fusion of two skyrmions (scale bar is 25 nm).

As indicated in the main text, when ramping down the magnetic field to 0 mT some magnetic skyrmions remain stable. supplementary fig. 6a displays a spin polarized dI/dU map recorded after ramping down the magnetic field from −150 mT to 0 mT. Tip was out-of-plane spin-polarized. On one of the narrow terraces (green dotted box) three skyrmions remain. supplementary fig. 6b and c show two dI/dU maps of this area recorded after two consecutive changes of the tip spin polarization. In (b), the tip was in-plane spin-

polarized. The three skyrmions appear with the same orientation, which confirms that the spin structure is chiral. In (c) the tip was unpolarized and the skyrmions were imaged via the TAMR effect. Note that the top skyrmion of the image merged with a fourth skyrmion in (a). Indeed it is frequently observed that a spin polarized tip in this system interacts with the spin structure. As an example, supplementary fig. 6d show 3 spin polarized dI/dU maps recorded consecutively on a same area. The tip scanned the surface from left to right starting from the top of the image when the green arrow point down and starting from the bottom of the image when the green arrow point up. When tip is scanning, skyrmions are moved in the same direction than the tip motion until it get close enough to the neighboring skyrmion and that skyrmion-skyrmion repulsion become stronger than tip-skyrmion attraction. In some case, the merge of two skyrmions was observed as this is shown in supplementary fig. 6e. The origin of this interaction tip-skyrmion is unclear; it can come from tip stray field, spin transfer torque or joule heating.

Supplementary Note 8. Magnetic phase diagram

In order to study the stability of the different phases, we minimized their total energies via spin dynamics simulations. We solve the Landau-Lifschitz-Gilbert equation using the extended Heisenberg model parametrized from our DFT calculation. supplementary fig. 6 shows the energy of the different phases as a function of the applied magnetic field with respect to the energy obtained for the spin spiral with $D_{ij} = 0.2$ meV/Co. As discussed above, when $D_{ij} = 0.2$ meV/Co, the ground state of the system at $B = 0$ T is FM with in-plane magnetization (green dashed line). $D = 0.3$ meV/Co is enough to stabilize a spin-spiral ground state for B lower than ≈150 mT (pink dotted line). When B further increases, the FM state aligns to the perpendicular magnetic field. The out of plane the magnetized FM state becomes more stable (green solid line). In the entire range of applied field, we have calculated the energy of isolated skyrmions (shown as red dashed lines). Isolated skyrmions are always metastable with respect to both the FM and the spin spiral states. The energy density differences are very small. For example, at $B = 130$ mT, the energy difference between the isolated skyrmion state and the FM state is of the order of 0.5 μeV/Co.

Supplementary Note 9. Determination of the skyrmion radii

As indicated in the main text, any stray field of the tip modifies the skyrmion structure or laterally moves it during scanning. Therefore, to determine the dependency of the skyrmion shape with magnetic field (Fig. 5b in the main text), a bare tungsten tip was used to image

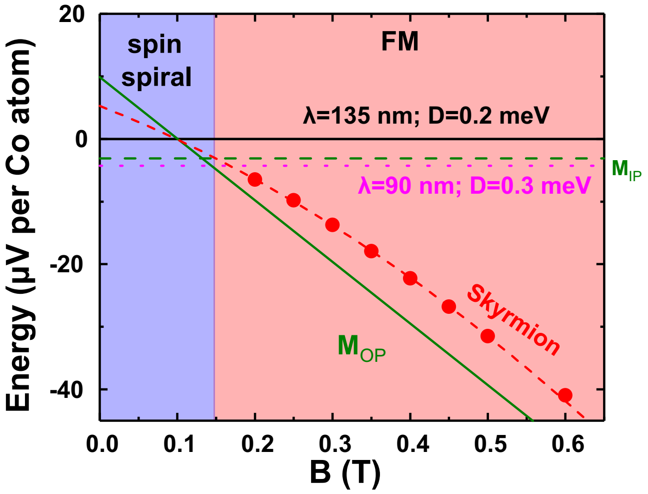

Supplementary Figure 7. **Magnetic phase diagram.** Energy of two spin spirals ($D = 0.2$ meV in black and $D = 0.3$ meV in magenta), isolated skyrmions and FM states as a function of the magnetic field B obtained via spin dynamics simulations. The origin of energy is set to the left rotating spin spiral energy minimum obtained via DFT calculations (black line). The period the spin spiral is found to be $\lambda = 135$ nm. The FM state is represented as a continuous green line when $\mathbf{M}$ is aligned perpendicular to the surface ($\mathbf{u}_z$ direction) and as a dashed line when $\mathbf{M}$ is in-plane. In the case of $D = 0.2$ meV/Co, the ground is FM as expected from DFT calculations. For $D = 0.3$ meV, the ground state is a spin spiral of period $\lambda = 90$ nm (magenta dotted line). The energy of isolated skyrmions is shown as dashed red line. When $B \in [150, 200]$ mT, the energy difference between the different magnetic states is as small as $2\,\mu$eV/Co. Isolated skyrmions are metastable in this region.

the skyrmion with the TAMR. In oder to take advantage of the high sensitivity of TAMR to distinguish between in-plane and out-of-plane oriented area, the definition of the skyrmion radius used here defer from the one use in [11]. We measured the diameter of the dark ring observed in the TAMR dI/dU maps (inset Fig. 5b - main text). The theoretical radius was obtained on the same way (green dot - Fig. 5b - main text). 8 show the theoretical skyrmion profiles of the magnetization orientation from the center to the edge for magnetic field from 200 to 600 mT. The skyrmion radius was determine by calculating the distance between the two opposite in-plane oriented sections i.e. two times the distance to the center represented by the vertical dashed lines.

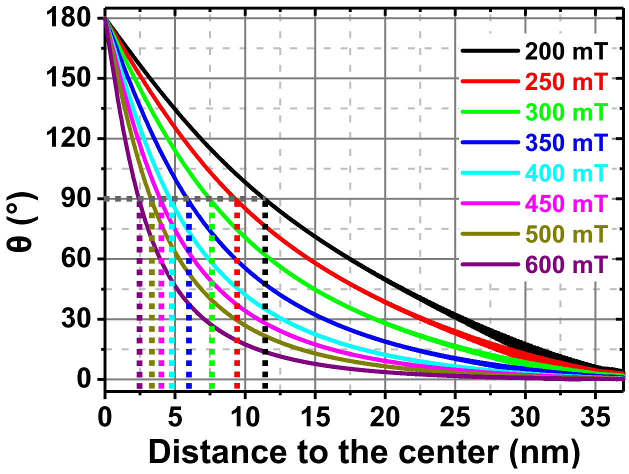

Supplementary Figure 8. **Determination of the skyrmion radii.** Magnetization orientation from the center to the edge for magnetic field from 200 to 600 mT of the calculated skyrmion profiles. Dashed vertical lines correspond to the distance to the center where magnetization is in-plane. This distance is half the skyrmion radius defined throughout this paper.

[1] Gabaly, F. E. *et al.* Structure and morphology of ultrathin Co/Ru(0001) films. *New Journal of Physics* **9**, 80 (2007).

[2] Rodary, G., Wedekind, S., Oka, H., Sander, D. & Kirschner, J. Characterization of tips for spin-polarized scanning tunneling microscopy. *Applied Physics Letters* **95**, 152513 (2009).

[3] Bode, M. *et al.* Magnetization-Direction-Dependent Local Electronic Structure Probed by Scanning Tunneling Spectroscopy. *Phys. Rev. Lett.* **89**, 237205 (2002).

[4] von Bergmann, K. *et al.* Tunneling anisotropic magnetoresistance on the atomic scale. *Phys. Rev. B* **86**, 134422 (2012).

[5] Gould, C. *et al.* Tunneling Anisotropic Magnetoresistance: A Spin-Valve-Like Tunnel Magnetoresistance Using a Single Magnetic Layer. *Phys. Rev. Lett.* **93**, 117203 (2004).

[6] Kurz, P., Förster, F., Nordström, L., Bihlmayer, G. & Blügel, S. Ab initio treatment of noncollinear magnets with the full-potential linearized augmented plane wave method. *Phys. Rev. B* **69**, 024415 (2004).

[7] Bruno, P. Tight-binding approach to the orbital magnetic moment and magnetocrystalline anisotropy of transition-metal monolayers. *Phys. Rev. B* **39**, 865–868 (1989).

[8] Zimmermann, B., Heide, M., Bihlmayer, G. & Blügel, S. First-principles analysis of a homochiral cycloidal magnetic structure in a monolayer Cr on W(110). *Physical Review B* **90**, 115427 (2014).

[9] Fert, A. & Levy, P. M. Role of anisotropic exchange interactions in determining the properties of spin-glasses. *Phys. Rev. Lett.* **44**, 1538–1541 (1980).

[10] El Gabaly, F. *et al.* Imaging Spin-Reorientation Transitions in Consecutive Atomic Co Layers on Ru(0001). *Phys. Rev. Lett.* **96**, 147202 (2006).

[11] Bogdanov, A. N. & Hubert, A. The properties of isolated magnetic vortices. *Phys. status solidi* **186**, 527–543 (1994).
